# DRD4 Rare Variants in Attention-Deficit/Hyperactivity Disorder (ADHD): Further Evidence from a Birth Cohort Study

**DOI:** 10.1371/journal.pone.0085164

**Published:** 2013-12-31

**Authors:** Luciana Tovo-Rodrigues, Luis A. Rohde, Ana M. B. Menezes, Guilherme V. Polanczyk, Christian Kieling, Julia P. Genro, Luciana Anselmi, Mara H. Hutz

**Affiliations:** 1 Genetics Department, Federal University of Rio Grande do Sul, Porto Alegre, Rio Grande do Sul, Brazil; 2 Child and Adolescent Psychiatric Division, Hospital de Clinicas de Porto Alegre, Federal University of Rio Grande do Sul, Porto Alegre, Rio Grande do Sul, Brazil; 3 National Institute of Developmental Psychiatry for Children and Adolescents (INCT-CNPq), Brazil; 4 Graduate Program in Epidemiology, Federal University of Pelotas, Pelotas, Rio Grande do Sul, Brazil; 5 Department of Psychiatry, Medical School and Research Support Center on Neurodevelopment and Mental Health, University of São Paulo, São Paulo, São Paulo, Brazil; Georgetown University, United States of America

## Abstract

The dopamine receptor D4 (DRD4) is one of the most studied candidate genes for Attention-Deficit/Hyperactivity Disorder (ADHD). An excess of rare variants and non-synonymous mutations in the VNTR region of 7R allele in ADHD subjects was observed in previous studies with clinical samples. We hypothesize that genetic heterogeneity in the VNTR is an important factor in the pathophysiology of ADHD. The subjects included in the present study are members of the 1993 Pelotas Birth Cohort Study (N=5,249). We conducted an association study with the 4,101 subjects who had DNA samples collected. The hyperactivity-inattention scores were assessed through the parent version of the Strengths and Difficulties Questionnaire at 11 and 15 years of age. The contribution of allele’s length and rare variants to high hyperactivity/inattention scores predisposition was evaluated by multivariate logistic regression. No effect of allele length was observed on high scores of hyperactivity-inattention. By contrast, when resequencing/haplotyping was conducted in a subsample, all 7R rare variants as well as non-synonymous 7R rare variants were associated with high hyperactivity/inattention scores (OR=2.561; P=0.024 and OR=3.216; P=0.008 respectively). A trend for association was observed with 4R rare variants. New coding mutations covered 10 novel motifs and many of them are previously unreported deletions leading to different stop codons. Our findings suggest a contribution of DRD4 7R rare variants to high hyperactivity-inattention scores in a population-based sample from a large birth cohort. These findings provide further evidence for an effect of DRD4 7R rare variants and allelic heterogeneity in ADHD genetic susceptibility.

## Introduction

Attention-deficit/hyperactivity disorder (ADHD) is characterized by impairing symptoms of inattention and/or hyperactivity and impulsivity [1]. This disorder has a heritability of approximately 76% [2], affecting about 5% of school-age children. It is a precursor of behavioral problems in adolescence and adulthood [3]. Among the dopamine-related genes investigated to date, the dopamine receptor D4 (DRD4) gene has been widely studied and it is the candidate gene with the strongest association with ADHD in different meta-analysis studies [4]. The most investigated polymorphism is a 48pb VNTR in its third exon. The alleles vary from two to eleven fold repeat (2R-11R), and the most prevalent are the 4R (65.1%), 7R(19.2%) and 2R(8.8%) [5]. Despite the evidences that 7R allele might be a susceptibility allele for ADHD, different groups have reported mixed results [4].

The VNTR region encodes a portion of the third cytoplasmic loop of Dopamine D4 Receptor which is considered an intrinsically disordered region of proteins. These regions lack a stable 3-D unique conformation and provide large interaction surface, conformational flexibility and exposure of short linear motifs, mediating the interaction with several partners [6]. Some properties described as important for signal transduction and proper protein functionality are usually associated to disordered regions, such as degradation (PEST) motifs, post-translational modifications and linear interaction motifs [7-9]. Dopamine D4 Receptor presents several SH3 motifs recognized in its third cytoplasmic loop [10]. Although their biological function within DRD4-VNTR has not been fully elucidated, this kind of domain-binding motif is widely described as important in cell–cell communication and signal transduction [11]. 

In addition to repeat number variation, rare variants inside the VNTR region were also described [12-16]. The combination of specific mutations results in different motifs (repeats) that are identified by numbers [12]. The haplotypes are composed by a specific combination of repeats (see 12-15 for more details, and Figure S1). 2R (1-4), 4R (1-2-3-4) and 7R (1-2-6-5-2-5-4) are the most frequent haplotypes on worldwide populations [12-15]. The other haplotypes are considered rare. A growing number of evidence suggests that rare haplotypes are an important risk factor to develop ADHD, but the literature addressing this topic is extremely scarce. Higher frequency of rare variants in ADHD probands compared to both control individuals [13] and children with autism [14] was reported. Recently, it has been documented that ADHD Brazilian subjects from a clinical sample presented an excess of rare variants as well as non-synonymous mutations compared to controls. It is noteworthy that these differences were restricted to 7R allele [16].

General-population studies have important advantages over case–control studies [17,18]. When the main objective is to explore rare variants variability and their contribution to complex diseases, this design represents a unique opportunity due to the large sample size. Another important aspect is that clinical cases do not represent the full spectrum of cases found in the general population for the majority of disease, especially for mental disorders [19]. Considering that, genetic findings on ADHD in clinical samples might not be representative of the general populations since ADHD represents only the most severe part of the phenotype and it might be determined by different genetic mechanisms than other portions of the inattentive and hyperactive/impulsive trait in the population [20]. Thus, it is of interest to assess the role of the DRD4 allele length variants as well as rare variants in ADHD etiology in the general population.

The aims of the current study are to investigate the association of DRD4 VNTR length and rare variants with an epidemiological measure of inattention and hyperactivity/impulsivity symptoms in a Brazilian birth cohort study. We also explore the impact of non-synonymous rare mutations in the ADHD etiology. 

## Results

Ten different DRD4 VNTR alleles with 2 to 11 tandem repeats were present in this study. The 4/4R was the most observed genotype in both hyperactivity/inattention low-score and high-score groups, followed by 4/7R genotype. The genotypic frequencies observed in the total population as well as in the low-score and high-score groups are shown in Table S1. To evaluate the effect of 7R allele in this sample, DRD4 VNTR genotypes were grouped into two categories: with and without 7-repeat allele. A logistic regression adjusted for the effect of gender and ethnicity was performed. Genotypes containing the 7R allele were not associated with high hyperactivity/inattention scores (Table 1).When an allelic model was tested, again no association was observed with high hyperactivity/inattention scores (Table S2).

**Table 1:** Frequency of DRD4-VNTR genotypes regarding presence or absence of 7R allele and rare variants and respective estimated odds ratios (OR) for high hyperactivity and inattention scores.

Model	Genotype	Low-score group n (%)^A^	High-score group n (%)^A^	OR (95% CI)	P-value^D^
VNTR allele length	7R carriers	1,047 (35.8%)	122 (36.0%)	1.029 (0.812-1.302)^B^	0.815
	Others	1,879 (64.2%)	217 (64%)	1	--
Sequences - Model 1 (Synonymous and non-synonymous included)	Presence of at least 1 copy of 4R rare variant	77 (15.0%)	14 (21.0%)	1.790 (0.901-3.557)^C^	0.096
	Presence of at least 1 copy of 7R rare variant	38 (7.4%)	9 (13.4%)	2.561 (1.133-5.790)^C^	0.024
	Common variants	398 (77.6%)	44 (65.6%)	1	--
Sequences - Model 2 (Only non-synonymous included)	Presence of at least 1 copy of 4R rare protein variant	61 (11.9%)	10 (14.9%)	1.593 (0.740-3.428)^C^	0.234
	Presence of at least 1 copy of 7R rare protein variant	30 (5.8%)	8 (11.9%)	3.216 (1.348-7.671)^C^	0.008
	Common variants	422 (82.3%)	49 (73.2%)	1	--

^A^ The analysis considering VNTR allele length comprised a sample size of 3,265 subjects (2,926 in low-score group and 339 in high-score group) while the analysis with the sequences comprised 580 subjects (513 in in low-score group and 67 in high-score group);

^B^ Logistic regression adjusted for gender and ethnicity;

^C^ Logistic regression adjusted for gender, ethnicity and alcohol consumption during pregnancy;

^D^ Significance level at 0.05

As rare variants inside the VNTR are hypothesized as being a possible confounder for usual DRD4 VNTR and ADHD association studies, all 2R(n=31) and 7R(n=175) and many randomly selected 4R(n=521) homozygous had the VNTR region sequenced, comprising a sample size of 727 individuals. 

**Figure 1:**
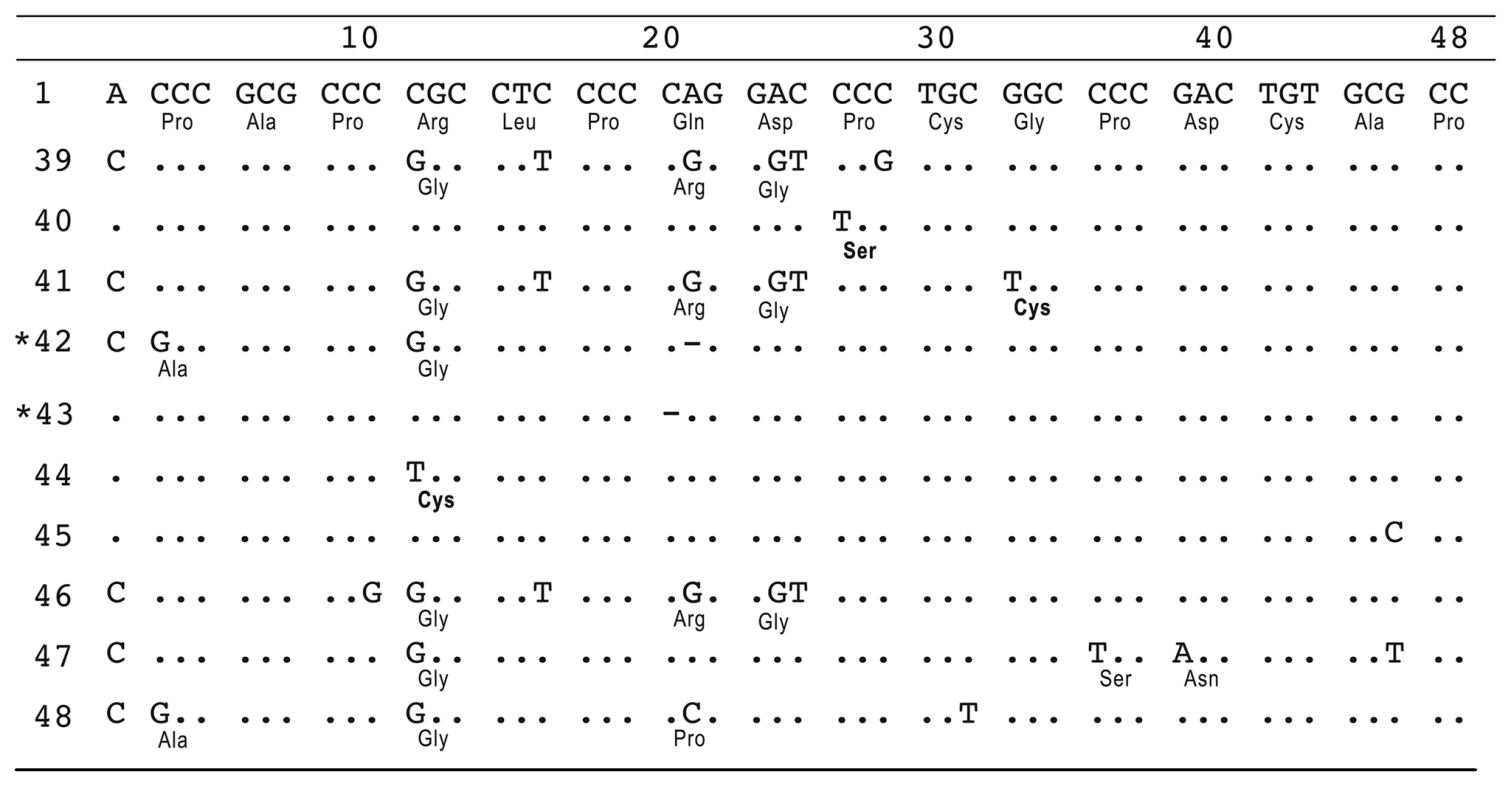
Nucleotide and amino acid sequences of the novel VNTR motifs. The nucleotide and corresponding amino acid (bold) sequences of 10 novel DRD4 exon3 48-bp repeat motifs are shown (repeats 39-48). * indicates 1bp deletion motifs.

**Figure 2:**
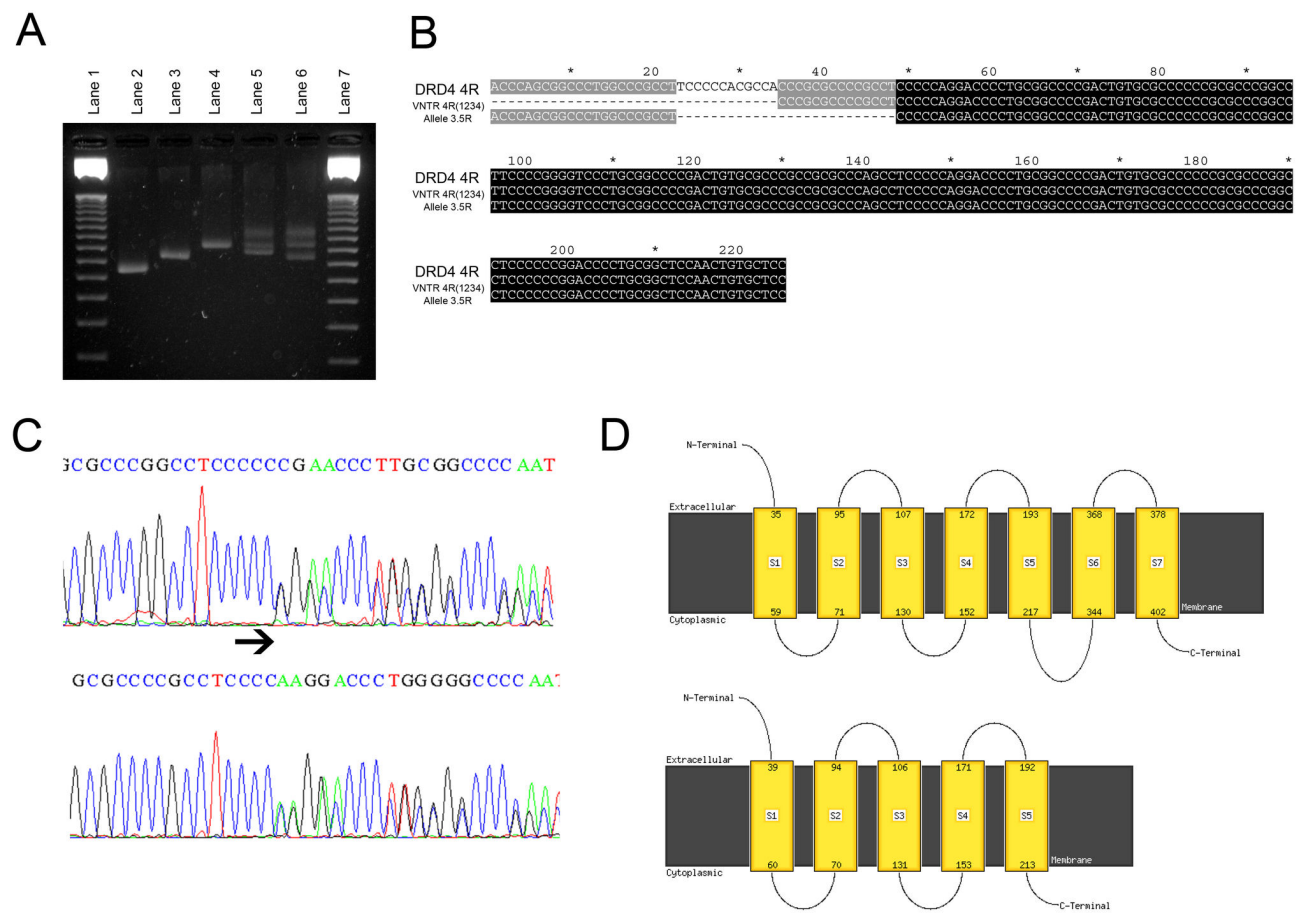
Characterization of DRD4 novel deletions. (A) PCR analysis of 3.5 allele. Lane 1: DNA 50bp marker, Lane 2: homozygote for 2 repeats; Lane 3 homozygote for 3 repeats; Lane 4: homozygote for 4 repeats; Lane 5: heterozygote for 3 and 4 repeats; Lane 6: heterozygote for 3.5 and 4 repeats. PCR products were run on a 3.5% agarose gel. A heteroduplex band is seen for heterozygotes in lanes 5 and 6. (B) The allele 3.5R alignment. The 3.5R refers to an allele with a length between 3 and 4 repeats. Sequencing of this size allele revealed a deletion of 26bp sequence (11bp immediate 5’ downstream sequence of the VNTR and 15bp inside the first repeat of VNTR) of the most common 4R haplotype. (C) Sequence chromatogram of DRD4-7R nucleotide deletions(arrow) at VNTR positions117 (del117C, above) and 20 (del20C, below). (D) Topology prediction of proteins encoded by VNTR with (below) and without (above) deletions. Schematic diagram of the transmembrane region (yellow). The deletions exclude the prediction of the two last transmembrane domains.

Considering the 1,454 sequenced alleles, we covered 30 different haplotypes: two at 2R allele, 15 at 4R and 13 at 7R alleles (Table 2, Figure S1). As expected, the most frequent haplotypes found in our general population sample were 2R (1-4), 4R (1-2-3-4) and 7R (1-2-6-5-2-5-4). A number of new coding SNPs covered 10 novel motifs (repeats from 39 to 48; Figure 1). Six novel haplotypes were found in 4R allele category and six in 7R. Among new motifs, we identified many unreported deletions. Two of them are1bp deletions at 7R allele. The first is a modification of a repeat previously identified as number 6 [12] where a deletion of a cytosine is observed at the 21^st^ base of the VNTR motif. This repeat was called 42. The other is a modification of motif number 1 where a deletion of a cytosine located at position 20 of the motif is found (repeat 43). The alignment of deleted motifs as well as their chromatograms can be seen on Figure 1, Figure 2 and Figure S1. These deletions lead to a frameshift with the occurrence of a premature stop codon at the position 393(M393X) instead of the position 468 in the protein. The other alteration comprises a 26bp deletion at the 4R allele (Figure 2). This deletion leads to a frameshift in which the stop codon UGA at position 420 is substituted by the codon for arginine. Translation of the protein thus continues until a stop codon encountered at codon 440, leading to a 20-residue-long C-terminal tail. In both cases, the two last transmembrane domains of D4 receptor would be drastically altered by incorporation of several disordered instead of hydrophobic residues, as shown by transmembrane topology prediction (Figure 2).

**Table 2:** Haplotypes observed in total sample, in low-score and high-score group sand their frequencies.

Haplotype per Allele length	Population sample	^A^Low-score group	^B^High-score group	Published populational data [12,16]	Published ADHD sample [13,16]
***2R Allele***	**N=62**	**N=50**	**N=0**	**N= 55**	**N=23**
1-4	61 (98.4%)	50 (100%)	0	43 (78.2%)	23 (100%)
30-4	1	0	0	12	0
***4R Allele***	**N=1,042**	**N=738**	**N=96**	**N=292**	**N=225**
1-2-3-4	912 (87.5%)	655 (88.8%)	82 (85.4%)	281 (96.2%)	211 (93.4%)
1-2-13-4	34	22	5	3	2
1-2-14-4	32	20	4	3	3
1-8-3-4	27	17	2	1	2
1-2-5-4	14	11	0	1	4
1-2-12-4	3	2	1	2	0
1-2-6-4	2	2	0	0	2
1-26-3-4	1	1	0	0	1
1-17-3-4	1	0	0	1	0
**1-2-14-47**	4	2	0	0	0
**1-2-48-4**	1	0	0	0	0
**1-46-13-4**	6	3	2	0	0
**45-2-3-4**	2	1	0	0	0
**44-2-3-4**	2	1	0	0	0
**Allele3.5R**	1	1	0	0	0
***7R Allele***	**N=350**	**N=238**	**N=38**	**N=218**	**N=110**
1-2-6-5-2-5-4	287 (82.0%)	197 (82.8%)	28 (73.7%)	206 (94.5%)	94 (85.5%)
1-2-6-5-2-5-19	15	10	1	5	3
1-8-25-5-2-5-4	14	10	3	2	3
1-2-3-17-2-5-4	10	6	1	2	3
1-2-6-1-2-3-4	8	4	2	0	4
1-2-6-5-2-3-4	6	5	1	3	2
**1-2-5-5-2-5-4**	3	1	1	0	0
1-2-6-5-37-5-4	1	1	0	0	1
**1-2-6-5-39-3-4**	1	0	0	0	0
**40-2-6-5-2-5-4**	1	1	0	0	0
**1-41-6-5-2-5-4**	1	1	0	0	0
**1-2-42-5-2-5-4**	1	1	0	0	0
**43-2-6-5-2-5-4**	2	1	1	0	0

The Haplotypes 1-4 and 30-4 Encode the Same Protein, the Haplotypes 1-2-14-4 and 45-2-3-4 Encode the Same Protein as 1-2-3-4. The Haplotype 1-2-6-5-2-5-19 Encodes the Same Protein as 1-2-3-5-6-5-4

^A^ SDQ hyperactivity-innatention≤7 at 11 and 15 years-old

^B^ SDQ hyperactivity-innatention≥8 at 11 and 15 years-old

In bold are the novel haplotypes found.

To evaluate the impact of rare variants in hyperactivity/inattentions scores, two models of multivariate logistic regressions were performed based on different strategies of pooling rare variants. The frequency of rare variants in case and control group is described in Table 2. 

As 7R rare variants are suggested to be associated to ADHD, rare variants were classified in specific 4R and 7R and included in the regression analysis as independent variables (first logistic regression model). This analysis revealed that individuals carrying at least one 7R rare haplotype were at a significantly increased risk for developing high hyperactivity/inattention scores compared with those homozygous for most common haplotypes (OR=2.561; P=0.024). A trend for association was observed for 4R rare variants (Table 1). 

As mutations that determine amino acid changes might be deleterious and potentially disease causing, we investigated the impact of non-synonymous substitution of rare variants in hyperactivity/inattention symptoms. For this analysis individuals were classified as carrying or not 4R or 7R variants that have at least one non-synonymous mutation. 4R rare haplotypes containing missense variants presented a trend in ADHD risk. On the other hand, carriers of 7R rare haplotypes containing missense variants had a greater than three-fold increase in the odds of developing higher hyperactivity/inattention scores as compared to those homozygous for the common haplotype (OR=3.216; P=0.008; Table 1). 

Many functional properties related to protein disorder were assessed in each haplotype. The results of potential alterations due to mutations are shown in table 3 and Figure S2. As expected, this region was predicted as to be highly disordered. Although the estimated mean disorder indexes are high and very similar for all haplotypes, many differences can be observed if the mutations are individually analyzed (Figure S2). Many of them confer disorder to order substitution on protein, such as R>C, G>C, G>V and P>Q. When the phosphorylation of serines, threonines and tyrosines were considered, the alterations were more obvious. Many mutations created potential phosphorylation sites in in two 7R (1-2-6-5-37-5-4) (Serine 74) and (40-2-6-5-2-5-4) (Serine 9) haplotypes. Regarding the potential sites for interaction and/or degradation PEST motifs were predicted in the 4R(1-17-3-4), 7R(1-8-25-5-2-5-4) and 7R(1-2-6-5-37-5-4) haplotypes. The most frequent haplotypes showed no potential sites for phosphorylation or potential PEST motifs. Moreover, many SH3 binding sites were described as disrupted in the following haplotypes: 4R(1-17-3-4) (aa 22-28), 7R(1-2-3-17-2-5-4) (aa 61-67) and 7R(40-2-6-5-2-5-4) (aa 3-9, 6-12). 

**Table 3:** Functional predictions related to disordered regions for observed haplotypes.

Haplotype per Allele length	^A^Disorder Index (Mean)	PEST motif EpestFind	^B^Phosphorylation (Residue)	^B^SH3 sites ELM (Position of missing sites)
***2R Allele***				
1-4	0.7411	Absence	No	5
30-4	0.7411	Absence	No	5
***4R Allele***				
1-2-3-4	0.851	Absence	No	13
1-2-13-4	0.848	Absence	No	13
1-2-14-4	0.851	Absence	No	13
1-8-3-4	0.810	Absence	No	13
1-2-5-4	0.857	Absence	No	13
1-2-12-4	0.848	Absence	No	13
1-2-6-4	0.856	Absence	No	13
1-26-3-4	0.842	Absence	No	13
1-17-3-4	0.858	Presence	No	12 (22-28)
**1-2-14-47**	0.834	Absence	No	13
**1-2-48-4**	0.856	Absence	No	13
**1-46-13-4**	0.848	Absence	No	13
**45-2-3-4**	0.851	Absence	No	13
**44-2-3-4**	0.819	Absence	No	13
**Allele3.5R**	--	--	--	--
***7R Allele***				
1-2-6-5-2-5-4	0.906	Absence	No	25
1-2-6-5-2-5-19	0.906	Absence	No	25
1-8-25-5-2-5-4	0.884	Presence	No	25
1-2-3-17-2-5-4	0.907	Absence	No	24 (61-67)
1-2-6-1-2-3-4	0.893	Absence	No	25
1-2-6-5-2-3-4	0.903	Absence	No	25
**1-2-5-5-2-5-4**	0.907	Absence	No	25
1-2-6-5-37-5-4	0.912	Presence	Yes (Ser74)	25
**1-2-6-5-39-3-4**	0.903	Absence	No	25
**40-2-6-5-2-5-4**	0.904	Absence	Yes (Ser9)	23 (3-9; 6-12)
**1-41-6-5-2-5-4**	0.874	Absence	No	25
**1-2-42-5-2-5-4**	--	--	--	--
**43-2-6-5-2-5-4**	--	--	--	--

^A^ The graphics showing individual disorder index for each amino acid are shown in Figure S2.

^B^ The positions of residues are related to VNTR peptide sequence showed in Figure S2

## Discussion

We reported herein the unusual architecture of DRD4 gene as well as an important association between high hyperactivity-inattention scores and rare variants, especially in 7R allele. Our analysis suggests that 7R rare variants might have a contribution to the multifactorial inheritance of ADHD while 7R length allele might not. 

Our results are in agreement with previous findings [12-16]. We observed a high variability inside DRD4 VNTR in the general population as reported elsewhere [12-15]. Also, a higher variability in the DRD4-VNTR region was observed in ADHD subjects when compared to controls [13] and to autistic subjects [14]. It is important to emphasize that all published results addressing this subject to date showed a consistent association between DRD4 rare variants, especially inside 7R allele, and ADHD [13,14,16].

An increasing body of evidence suggests that rare variants may play a role in complex diseases, including neuropsychiatric traits. To date the Genome Wide Association (GWA) studies conducted on ADHD samples were not able to find any common variants significantly associated with the disorder. Although the small sample size involving these studies is pointed as a possible limitation to find a significant association at genomic level, “synthetic” signals of association on GWA in other case-control studies are reported [21]. Then, the common-disease-rare-variant (CDRV) hypothesis is an attractive framework to understand the susceptibility of the disorder. This hypothesis proposes that the sum of the effects of a series of low frequency variants, each conferring a moderate but readily detectable increase in relative risk, compose an important proportion of the overall genetic risk for common diseases etiology [22]. Recent findings have drawn attention to the involvement of rare structural variants in the pathophysiology of ADHD [23]. Point mutations inside DRD4 appear to be another important example of this phenomenon.

Our analysis suggests that rare variants may make a contribution to the multifactorial inheritance of common diseases. Thus, even though individual rare variants may not contribute much to the overall inherited tendency of a disease, their discovery is likely to be important for understanding disease etiology. Another important aspect of this study is its focus on the identification of multiple rare variations, since individuals with ADHD might frequently possess any of them. This suggests that all the variations contributing to the pool of variations that were greater in frequency among the individuals with the disease phenotype might perturb D4 receptor activity. 

Many studies already addressed the plausibility of studying the impact of mutations in disordered regions [24,25]. The main hypothesis is that altering the disorder status in proteins could affect many important biological processes [25]. In this study we observed that many mutations are able to perturb some disorder-related properties. Differences in disorder profile, PEST motifs recognition, additional phosphorylation sites and disruption of binding sites could affect D4 receptor functionality. Among the possible impaired functions there are reduction of loop flexibility, facilitated recognition for internalization and degradation, unusual desensitization, homo-heterodimerization disparities, and disruption of usual interactions. Further functional studies as well as studies evaluating DRD4 variability in other psychiatric disorders are needed to better understand the role of these mutations in disease etiology.

This is the first study showing potential null alleles due to deletions inside the VNTR. In humans, only one homozygous for the null allele was reported [26]. Although the subject did not present ADHD or other psychiatric disorder, many symptoms possibly related to the absence of DRD4 protein were observed, such as somatic ailments, obesity and disturbances of the autonomic nervous system [26]. Out of four subjects containing deletions presented here, only one had extreme phenotypes. However, all subjects are heterozygotes. Further careful phenotype assessment of subjects carrying null alleles is needed to better clarify the role of the DRD4 gene in normal and pathologic development. 

Our results must be interpreted in the context of some limitations. Although the hyperactivity/Inattention subscale of the SDQ shows high diagnostic performance for ADHD, the use of this epidemiological instrument might have created biases in our phenotypic definition. However, it is important to note that our analyses were based on individuals who were classified in the same group – low or high score groups – in both assessments. So, we are confident that we selected those individuals in the population with deviant and stable inattentive/hyperactive profile. Second, no genomic control was performed. Therefore our findings might have been biased by hidden genetic heterogeneity present in our specific sample of the southern Brazilian population. However, our case and control groups contain a similar ethnic distribution and the analysis was adjusted for ethnicity. Moreover, it is important to note that recent studies using different sets of informative markers did not show heterogeneity across main Brazilian populations [27] or significant population structure in southern Brazilians [28]. Finally, since the 10 novel motifs found could be artifacts, we re-sequenced all samples in which they were observed through different PCR products to confirm mutations. 

In conclusion, DRD4 7R rare variants are associated with hyperactivity and inattention at the general population level confirming previous studies on ADHD probands. These results indicate that the DRD4 7R rare variants could play an important role in ADHD genetic susceptibility. *In silico* analysis of putative effect of these mutations on receptor function suggests that they could have an impact on receptor signaling, degradation, internalization and desensitization. In addition, we document that taking in consideration the sequence of DRD4 VNTR is very important to estimate the contribution of this dopaminergic receptor to the pathophysiology of ADHD.

## Material and Methods

### Ethics Statement

The project was approved by the Institutional Review Board of the School of Medicine, Federal University in Pelotas (UFPel). Parents or guardians signed an informed consent form authorizing their own participation and that of the children in the study.

### Subjects

Subjects included in the present study were members of the 1993 Pelotas Birth Cohort Study [29,30], a birth cohort carried out in the municipality of Pelotas, Southern Brazil in 1993. This article draws on data collected at the perinatal visit and the 11-year and 15-year follow-up visits. From all live births in the city of Pelotas in Brazil (N=5,249), 87.5% and 85.7% of them were respectively re-assessed at 11 and 15 years of age (for more details, see Figure S3). 

### Phenotypic Assessments

To evaluate attention and hyperactivity/impulsive problems, mothers or guardians answered the Strengths and Difficulties Questionnaire (SDQ) [31]. The SDQ is one of the most commonly used and validated instruments to assess child mental health in epidemiological samples [32,33]. It comprises 25 questions and allows computation of raw scores for five sub-scales with five questions each: emotional symptoms, conduct problems, hyperactivity (and inattention) symptoms, problems with peer relations, and pro-social behavior problems. Our outcome variable was the sub-scale of attention/hyperactivity problems from the parents’ version of SDQ [34]. 

### DNA Collection and Genotyping

Out of the total sample size, 4,101 subjects had the DNA collected and were genotyped for the VNTR polymorphism. Saliva DNA of the adolescents was isolated using the Oragene DNA kit (DNA GenotekInc Ontario, Canada) according to the manufacturer’s instructions. For amplification of the VNTR in DRD4 exon III, D4-3 and D4-42 primers described previously were used [15]. PCRs were conducted in 25-μl volumes containing 40ng of genomic DNA, 5% DMSO, 2mM MgCl_2_, 200μM dATP, dCTP, dTTP, 100μM dGTP, 100 μM 7-deaza-dGTP, 1.2μM of each primer, 1× PCR buffer (Promega), and 1 unit of Go*Taq*HotStart DNA polymerase (Promega). Amplification was performed by using Veriti™ Thermal Cyclers (Applied Biosystems). A 2-min, 94°Cinitial denaturation was used followed by 35 cycles of 95°C for 30 sec, 62°C for 30 sec and 72°C for 40 sec. The final step was annealing of 74°C for 4 min. Out of 4,101 amplified subjects, all 2R and 7R and random 521 4R homozygous were selected to have the VNTR region sequenced, comprising a sample size of 727 individuals. One 2R and seven 7R homozygous subjects failed to have their DRD4 VNTR sequenced after three times and were excluded from subsequent analysis. The PCR products consisted in fragments of 379bp for 2R allele, 475bp for 4R allele and 619bp for 7R allele. These amplicons were purified by enzymatic treatment before sequencing as reported [12]. For the individual carrying the 3.5R allele, the two allelic PCR products (3.5R and 4R alleles) were isolated and then both alleles were sequenced. The sequencing procedure and primers used were previously reported [16]. When new mutations were observed or when sequences were dubious, the samples were resequenced through independent PCR products. DNA sequences of the novel *DRD4* haplotypes reported in this paper have been submitted to GenBank (Accession numbersJQ064942-JQ064953).

### Sequences and Bioinformatic Analysis

Sequence analyses were accomplished using CodonCode Aligner v3.0.1 software (Codon Code Corporation, Dedham, MA). Haplotypes were estimated using a Bayesian method implemented in PHASE2.1 software [35]. The haplotypes were derived according to the motifs and point mutations as described previously [12]. Transmembrane topology prediction was provided by MEMSAT3algorithm [36] available via the PSIPRED server (http://bioinf.cs.ucl.ac.uk/psipred/). To access the possible effect of mutations in the protein, we analyzed the sequences considering different parameters. We estimated the mean disorder index for each predicted peptide using PONDR^®^ VT-LX predictor (www.pondr.com) [37]. PEST motif is a classical protein degradation targeting signal enriched in proline (P), glutamic acid (E), serine (S) and threonine (T) and confers rapid instability to proteins. Presence or absence of this motif was evaluated through ePESTfind server (http://emboss.bioinformatics.nl/cgi-bin/emboss/epestfind) [7]. Presence of unusual phospohorylation sites using NetPhos 2.0 server (http://www.cbs.dtu.dk/services/NetPhos/) [38], and prediction of SH3 binding sites using ELM server (http://elm.eu.org/) [39]. Access to PONDR® was provided by Molecular Kinetics. VL-XT is copyright©1999 by the WSU Research Foundation, all rights reserved. PONDR® is copyright©2004 by Molecular Kinetics, all rights reserved.

### Statistical Analysis

Our dependent variable is defined as a SDQ attention and hyperactivity score ≥ 8 in both assessments at 11 and 15 years of age. The rationale for this method of dichotomizing high vs. low hyperactivity/inattention scores in the current sample is described elsewhere [40]. Briefly, in order to assure that we were dealing with a clear deviant and stable phenotype, sample dichotomization in high (score > 8) and low (score < 7) hyperactivity/inattention scores in the SDQ hyperactivity-inattention subscale at both 11 and 15 years old was established according to previous analysis of ADHD in this sample. This threshold showed adequate diagnostic performance for ADHD [40]. All phenotype definitions were done a priori of any analyses. 

A binary variable indicating whether subjects had “high hyperactivity/inattention score” was used as the dependent variable in logistic regression analyses. The first analysis focused on the association between the genotypes with DRD4 7R allele (risk allele), and presence of high hyperactivity/inattention score. Out of the initial sample size (4,101 subjects), 3,265 subjects were included in the analysis. Following the cut-off criteria mentioned above, those subjects that presented discordant hyperactivity/inattention scores at 11 and 15 were excluded from the analysis (Figure S3). Subsequent analyses included the subsample selected for VNTR sequencing (out of the 727 sequenced samples, 580 individuals had concordant hyperactivity/inattention scores at both assessments and, thus, were included in the analyses). To exclude the effect of genotype in the subsample, a multivariate logistic regression considering the allele length as main effect adjusted for gender and ethnicity was performed. As no association was observed (Table S3), the participants were grouped according to genetic variation in the DRD4-VNTR sequence following two strategies: 1) carriers and non-carriers of 4R or 7R rare variants and 2) carriers and non-carriers of 4R or 7R rare variants with missense mutations. Thus, two independent models were constructed to assess potential odds ratio for high scores of attention and hyperactivity in carriers of 4R or 7R rare variants and carriers of 4R or 7R rare variants with non-synonymous mutations with 95% confidence intervals (95% CI) using multivariate logistic regression with adjustment for potential confounders. These groups were compared to common variant carriers. Potential confounders (gender, alcohol consumption during pregnancy and ethnicity) comprise a priori selected variables based on conceptual analyses of the literature and on a statistical definition (association with the main factor and with the outcome at P< 0.10). Statistical significance was defined as a two-tailed P-value < 0.05. Statistical analysis was performed using PASW Statistics 18, Release Version18.0.0 (SPSS, Inc., 2009,Chicago, IL, USA, www.spss.com). 

In this study, the term ‘‘motif’’ refers to a variant sequence of the VNTR 48bp repeat unit. A haplotype means a specific motif composition, and allele refers to a specific length variant. Haplotype nomenclature and the criteria to define rare haplotypes were those previously described [12-14]. 

## Supporting Information

Figure S1 **2R, 4R and 7R haplotype sequences alignment.**
(PDF)

Figure S2 **Protein sequences alignment and disorder prediction graphics for every 4R and 7R haplotypes.**
(PDF)

Figure S3 **Study flow chart specifying the sample size for each analysis.**
(PDF)

Table S1 **Frequencies of genotypes considering allele length observed in total sample, in low-score and high-score groups.**
(DOCX)

Table S2 **Allele frequency model for 4R and 7R.**
(DOCX)

Table S3 **Frequency of DRD4-VNTR genotypes regarding presence or absence of 7R allele length and respective estimated odds ratios (OR) for high hyperactivity and inattention scores in the sequenced subsample.**
(DOC)
