# Supplementary material for: DRD4 Rare Variants in Attention-Deficit/Hyperactivity Disorder (ADHD): Further Evidence from a Birth Cohort Study
Source: PLoS One. 2013 Dec 31;8(12):e85164. doi: 10.1371/journal.pone.0085164 (PMC3877354; doi:10.1371/journal.pone.0085164)
Supplement: Figure S1 — 2R, 4R and 7R haplotype sequences alignment. (PDF) [file pone.0085164.s001.pdf]

## 2R haplotypes

```
                        1st motif                         48                2nd motif                        96
1-4    1 : ACCCGCGCCCCGCCTCCCCCAGGACCCCTGCGGCCCCGACTGTGCGCCCCCCGCGCCCGGCCTCCCCCCGGACCCCTGCGGCTCCAACTGTGCTCC : 96
30-4   1 : ..............................T................................................................. : 96
```

## 4R haplotypes

```
                                  1st motif                          48                     2nd motif                        96                    3rd motif                       144
1-2-3-4      1 : ACCCGCGCCCCGCCTCCCCCAGGACCCCTGCGGCCCCGACTGTGCGCCCCCCGCGCCCGGCCTTCCCCGGGGTCCCTGCGGCCCCGACTGTGCGCCCGCCGCGCCCAGCCTCCCCCAGGACCCCTGTGGCCCCGACTGTGCGCC : 144
1-2-13-4     1 : ..........................................................................................................G...................C................. : 144
1-2-14-4     1 : ..............................................................................................................................C................. : 144
1-8-3-4      1 : ..........................................................T..................................................................................... : 144
1-2-5-4      1 : .................................................................................................C........G...................C................. : 144
1-2-12-4     1 : ..........................................................................................................G..................................... : 144
1-2-6-4      1 : ..........................................................................................................G.........C.........C................. : 144
1-26-3-4     1 : .......................................................................T........................................................................ : 144
1-17-3-4     1 : ...............................................................C....C..AC.........T..A.......................................................... : 144
1-2-14-47    1 : ..............................................................................................................................C................. : 144
1-2-48-4     1 : ..........................................................................................................G.........C........................... : 144
1-46-13-4    1 : .........................................................G................................................G...................C................. : 144
45-2-3-4     1 : .............................................C.................................................................................................. : 144
44-2-3-4     1 : ..........T..................................................................................................................................... : 144
```

```
                        4th motif                        192
1-2-3-4      145 : CCCCGCGCCCGGCCTCCCCCCGGACCCCTGCGGCTCCAACTGTGCTCC : 192
1-2-13-4     145 : ................................................ : 192
1-2-14-4     145 : ................................................ : 192
1-8-3-4      145 : ................................................ : 192
1-2-5-4      145 : ................................................ : 192
1-2-12-4     145 : ................................................ : 192
1-2-6-4      145 : ................................................ : 192
1-26-3-4     145 : ................................................ : 192
1-17-3-4     145 : ................................................ : 192
1-2-14-47    145 : ....................A........................... : 192
1-2-48-4     145 : ................................................ : 192
1-46-13-4    145 : ................................................ : 192
45-2-3-4     145 : ................................................ : 192
44-2-3-4     145 : ................................................ : 192
```

7R haplotypes

```
                                    1st motif                                    48         2nd motif                                    96         3rd motif                                    144
1-2-6-5-2-5-4     1 : ACCCGCGCCCCGCCTCCCCCAGGACCCCTGCGGCCCCGACTGTGCGCCCCCCGCGCCCGGCCTTCCCCGGGGTCCCTGCGGCCCCGACTGTGCGCCCGCCGCGCCCGGCCTCCCCCCGGACCCCTGCGGCCCCGACTGTGCGCC : 144
1-2-6-5-2-5-19    1 : ................................................................................................................................................ : 144
1-8-25-5-2-5-4    1 : ..........................................................T...............................................A..................................... : 144
1-2-3-17-2-5-4    1 : ..........................................................................................................A.........A.........T................. : 144
1-2-6-1-2-3-4     1 : ................................................................................................................................................ : 144
1-2-6-5-2-3-4     1 : ................................................................................................................................................ : 144
1-2-5-5-2-5-4     1 : .................................................................................................C..................A........................... : 144
1-2-6-5-37-5-4    1 : ................................................................................................................................................ : 144
1-2-6-5-39-3-4    1 : ................................................................................................................................................ : 144
40-2-6-5-2-5-4    1 : .........................T.............................................................................................................................. : 144
1-41-6-5-2-5-4    1 : ...............................................................................T................................................................ : 144
1-2-42-5-2-5-4    1 : ....................................................................................................................-........................... : 143
43-2-6-5-2-5-4    1 : ...................-............................................................................................................................ : 143

                                    4th motif                                    192        5th motif                                    240        6th motif                                    288
1-2-6-5-2-5-4   145 : CCCCGCGCCCGGCCTCCCCCAGGACCCCTGCGGCCCCGACTGTGCGCCCCCCGCGCCCGGCCTTCCCCGGGGTCCCTGCGGCCCCGACTGTGCGCCCCCCGCGCCCGGCCTCCCCCAGGACCCCTGCGGCCCCGACTGTGCGCC : 288
1-2-6-5-2-5-19  145 : ................................................................................................................................................ : 288
1-8-25-5-2-5-4  145 : ................................................................................................................................................ : 288
1-2-3-17-2-5-4  145 : ....................C.............T..A.......................................................................................................... : 288
1-2-6-1-2-3-4   145 : A.........C......................................................................................G........A...................T................. : 288
1-2-6-5-2-3-4   145 : .................................................................................................G........A...................T................. : 288
1-2-5-5-2-5-4   145 : ................................................................................................................................................ : 288
1-2-6-5-37-5-4  145 : .............................................................................CG................................................................. : 288
1-2-6-5-39-3-4  145 : ...........................................................................G.....................G........A...................T................. : 288
40-2-6-5-2-5-4  145 : ................................................................................................................................................ : 288
1-41-6-5-2-5-4  145 : ................................................................................................................................................ : 288
1-2-42-5-2-5-4  144 : ................................................................................................................................................ : 287
43-2-6-5-2-5-4  144 : ................................................................................................................................................ : 287

                                    7th motif                                    336
1-2-6-5-2-5-4   289 : CCCCGCGCCCGGCCTCCCCCCGGACCCCTGCGGCTCCAACTGTGCTCC : 336
1-2-6-5-2-5-19  289 : .........T...................................... : 336
1-8-25-5-2-5-4  289 : ................................................ : 336
1-2-3-17-2-5-4  289 : ................................................ : 336
1-2-6-1-2-3-4   289 : ................................................ : 336
1-2-6-5-2-3-4   289 : ................................................ : 336
1-2-5-5-2-5-4   289 : ................................................ : 336
1-2-6-5-37-5-4  289 : ................................................ : 336
1-2-6-5-39-3-4  289 : ................................................ : 336
40-2-6-5-2-5-4  289 : ................................................ : 336
1-41-6-5-2-5-4  289 : ................................................ : 336
1-2-42-5-2-5-4  288 : ................................................ : 335
43-2-6-5-2-5-4  288 : ................................................ : 335
```
