# Supplementary material for: DRD4 Rare Variants in Attention-Deficit/Hyperactivity Disorder (ADHD): Further Evidence from a Birth Cohort Study
Source: PLoS One. 2013 Dec 31;8(12):e85164. doi: 10.1371/journal.pone.0085164 (PMC3877354; doi:10.1371/journal.pone.0085164)
Supplement: Figure S2 — Protein sequences alignment and disorder prediction graphics for every 4R and 7R haplotypes. (PDF) [file pone.0085164.s002.pdf]

Alignment of 4R amino acid sequence variants

```
                                16              32              48              64
1-2-3-4    : PAPRLPQDPCGPDCAPPAPGLPRGPCGPDCAPAAPSLPQDPCGPDCAPPAPGLPPDPCGSNCAP : 64
1-2-13-4   : PAPRLPQDPCGPDCAPPAPGLPRGPCGPDCAPAAPGLPQDPCGPDCAPPAPGLPPDPCGSNCAP : 64
1-2-14-4   : PAPRLPQDPCGPDCAPPAPGLPRGPCGPDCAPAAPSLPQDPCGPDCAPPAPGLPPDPCGSNCAP : 64
1-8-3-4    : PAPRLPQDPCGPDCAPPAPCLPRGPCGPDCAPAAPSLPQDPCGPDCAPPAPGLPPDPCGSNCAP : 64
1-2-5-4    : PAPRLPQDPCGPDCAPPAPGLPRGPCGPDCAPPAPGLPQDPCGPDCAPPAPGLPPDPCGSNCAP : 64
1-2-12-4   : PAPRLPQDPCGPDCAPPAPGLPRGPCGPDCAPAAPGLPQDPCGPDCAPPAPGLPPDPCGSNCAP : 64
1-2-6-4    : PAPRLPQDPCGPDCAPPAPGLPRGPCGPDCAPAAPGLPPDPCGPDCAPPAPGLPPDPCGSNCAP : 64
1-26-3-4   : PAPRLPQDPCGPDCAPPAPGLPRVPCGPDCAPAAPSLPQDPCGPDCAPPAPGLPPDPCGSNCAP : 64
1-17-3-4   : PAPRLPQDPCGPDCAPPAPGLPPDPCGSNCAPAAPSLPQDPCGPDCAPPAPGLPPDPCGSNCAP : 64
1-2-14-47  : PAPRLPQDPCGPDCAPPAPGLPRGPCGPDCAPAAPSLPQDPCGPDCAPPAPGLPQDPCGSNCAP : 64
1-2-48-4   : PAPRLPQDPCGPDCAPPAPGLPRGPCGPDCAPAAPGLPPDPCGPDCAPPAPGLPPDPCGSNCAP : 64
1-46-13-4  : PAPRLPQDPCGPDCAPPAPGLPRGPCGPDCAPAAPGLPQDPCGPDCAPPAPGLPPDPCGSNCAP : 64
45-2-3-4   : PAPRLPQDPCGPDCAPPAPGLPRGPCGPDCAPAAPSLPQDPCGPDCAPPAPGLPPDPCGSNCAP : 64
44-2-3-4   : PAPCLPQDPCGPDCAPPAPGLPRGPCGPDCAPAAPSLPQDPCGPDCAPPAPGLPPDPCGSNCAP : 64
```

Alignment of 7R amino acid sequence variants

```
                                      16                32                48                64                80
1-2-6-5-2-5-4  : PAPRLPQDPCGPDCAPPAPGLPRGPCGPDCAPAAPGLPPDPCGPDCAPPAPGLPQDPCGPDCAPPAPGLPRGPCGPDCAPPAPGLPQDP : 89
1-2-6-5-2-5-19 : PAPRLPQDPCGPDCAPPAPGLPRGPCGPDCAPAAPGLPPDPCGPDCAPPAPGLPQDPCGPDCAPPAPGLPRGPCGPDCAPPAPGLPQDP : 89
1-8-25-5-2-5-4 : PAPRLPQDPCGPDCAPPAPCLPRGPCGPDCAPAAPSLPPDPCGPDCAPPAPGLPQDPCGPDCAPPAPGLPRGPCGPDCAPPAPGLPQDP : 89
1-2-3-17-2-5-4 : PAPRLPQDPCGPDCAPPAPGLPRGPCGPDCAPAAPSLPQDPCGPDCAPPAPGLPPDPCGSNCAPPAPGLPRGPCGPDCAPPAPGLPQDP : 89
1-2-6-1-2-3-4  : PAPRLPQDPCGPDCAPPAPGLPRGPCGPDCAPAAPGLPPDPCGPDCAPPAPRLPQDPCGPDCAPPAPGLPRGPCGPDCAPAAPSLPQDP : 89
1-2-6-5-2-3-4  : PAPRLPQDPCGPDCAPPAPGLPRGPCGPDCAPAAPGLPPDPCGPDCAPPAPGLPQDPCGPDCAPPAPGLPRGPCGPDCAPAAPSLPQDP : 89
1-2-5-5-2-5-4  : PAPRLPQDPCGPDCAPPAPGLPRGPCGPDCAPPAPGLPQDPCGPDCAPPAPGLPQDPCGPDCAPPAPGLPRGPCGPDCAPPAPGLPQDP : 89
1-2-6-5-37-5-4 : PAPRLPQDPCGPDCAPPAPGLPRGPCGPDCAPAAPGLPPDPCGPDCAPPAPGLPQDPCGPDCAPPAPGLPRGPSGPDCAPPAPGLPQDP : 89
1-2-6-5-39-3-4 : PAPRLPQDPCGPDCAPPAPGLPRGPCGPDCAPAAPGLPPDPCGPDCAPPAPGLPQDPCGPDCAPPAPGLPRGPCGPDCAPAAPSLPQDP : 89
40-2-6-5-2-5-4 : PAPRLPQDSCGPDCAPPAPGLPRGPCGPDCAPAAPGLPPDPCGPDCAPPAPGLPQDPCGPDCAPPAPGLPRGPCGPDCAPPAPGLPQDP : 89
1-41-6-5-2-5-4 : PAPRLPQDPCGPDCAPPAPGLPRGPCCPDCAPAAPGLPPDPCGPDCAPPAPGLPQDPCGPDCAPPAPGLPRGPCGPDCAPPAPGLPQDP : 89
```

```
                        96               112
1-2-6-5-2-5-4  : CGPDCAPPAPGLPPDPCGSNCAP : 112
1-2-6-5-2-5-19 : CGPDCAPPAPGLPPDPCGSNCAP : 112
1-8-25-5-2-5-4 : CGPDCAPPAPGLPPDPCGSNCAP : 112
1-2-3-17-2-5-4 : CGPDCAPPAPGLPPDPCGSNCAP : 112
1-2-6-1-2-3-4  : CGPDCAPPAPGLPPDPCGSNCAP : 112
1-2-6-5-2-3-4  : CGPDCAPPAPGLPPDPCGSNCAP : 112
1-2-5-5-2-5-4  : CGPDCAPPAPGLPPDPCGSNCAP : 112
1-2-6-5-37-5-4 : CGPDCAPPAPGLPPDPCGSNCAP : 112
1-2-6-5-39-3-4 : CGPDCAPPAPGLPPDPCGSNCAP : 112
40-2-6-5-2-5-4 : CGPDCAPPAPGLPPDPCGSNCAP : 112
1-41-6-5-2-5-4 : CGPDCAPPAPGLPPDPCGSNCAP : 112
```

Intrinsic protein disorder prediction for 4R haplotypes

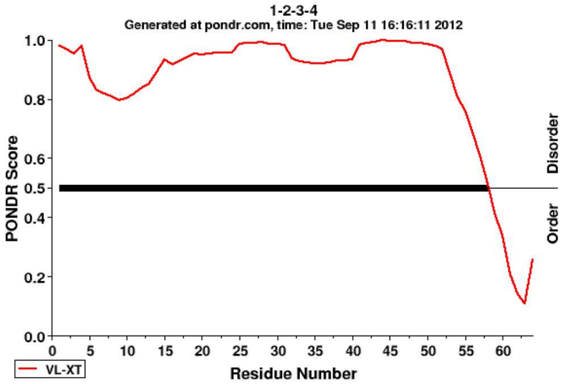

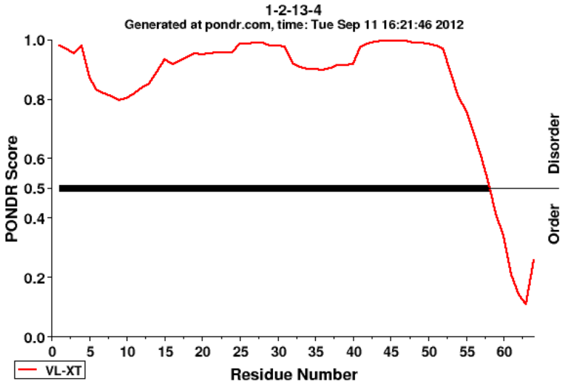

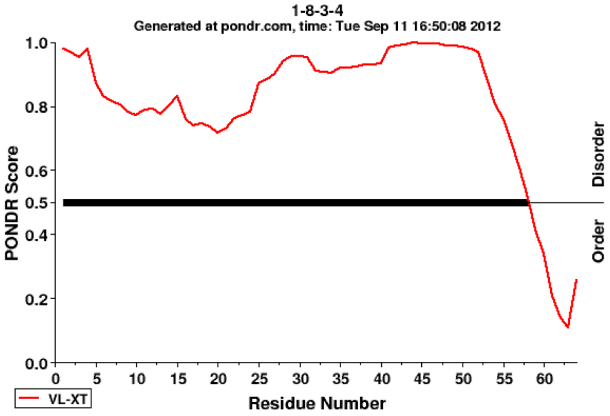

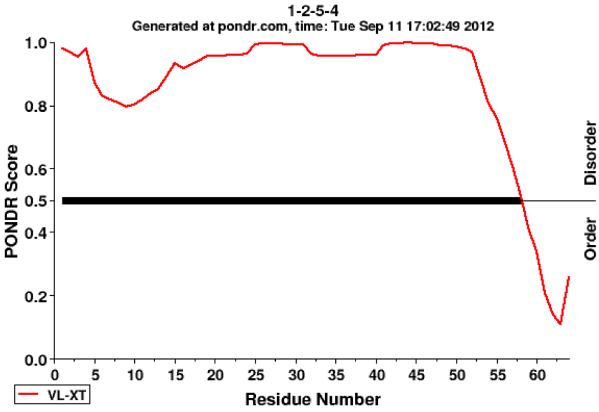

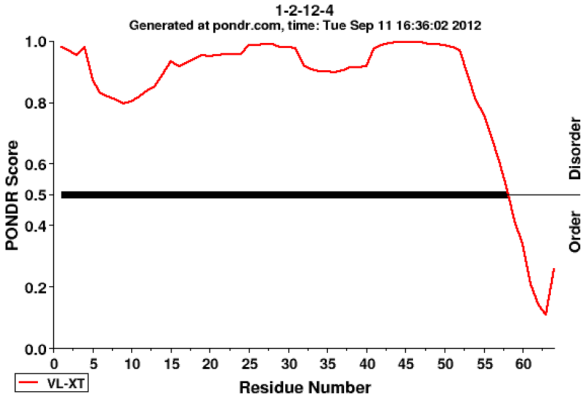

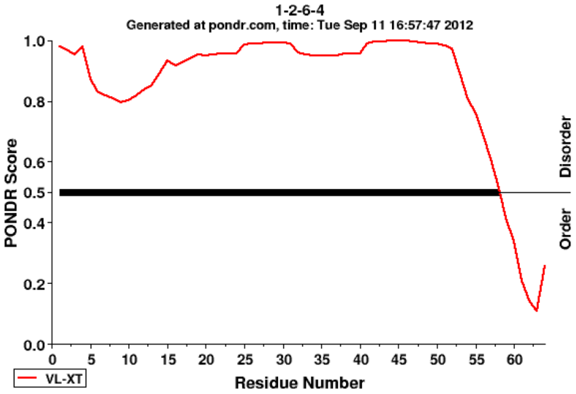

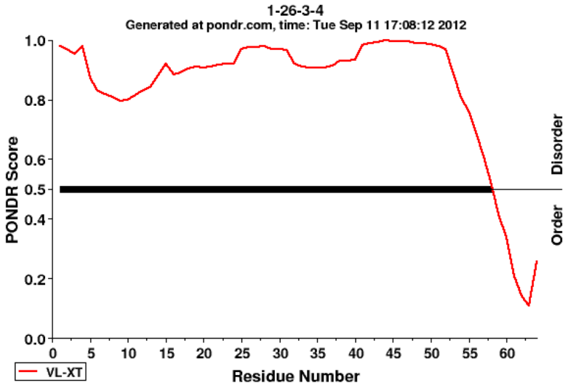

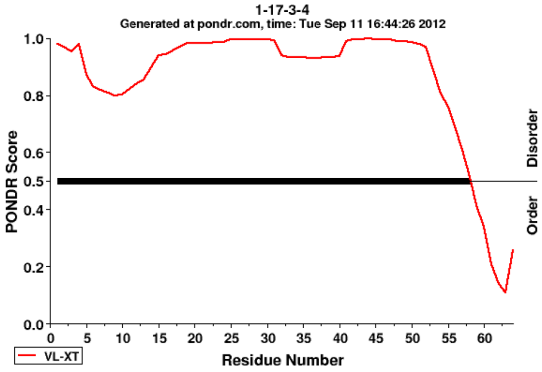

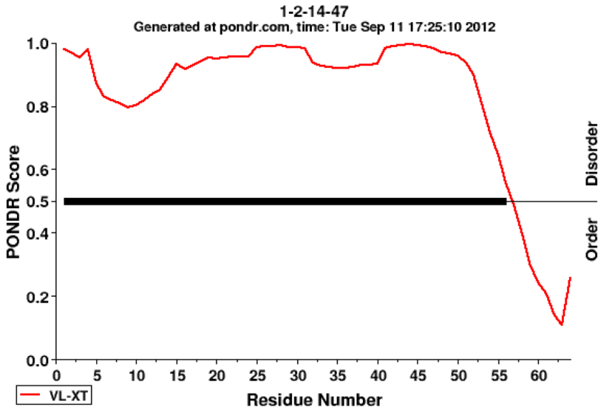

1-2-14-47
Generated at pondr.com, time: Tue Sep 11 17:25:10 2012
PONDR Score
Residue Number
Disorder
Order
VL-XT

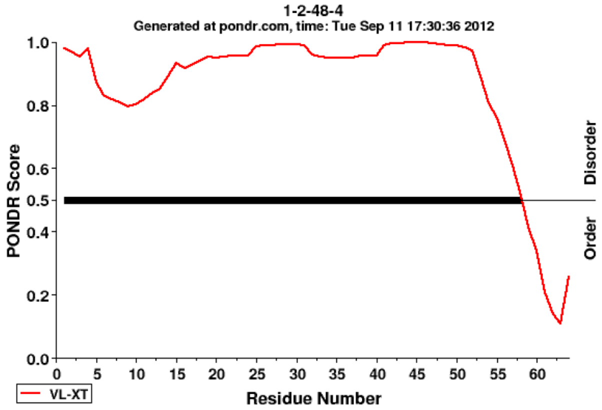

1-2-48-4
Generated at pondr.com, time: Tue Sep 11 17:30:36 2012
PONDR Score
Residue Number
Disorder
Order
VL-XT

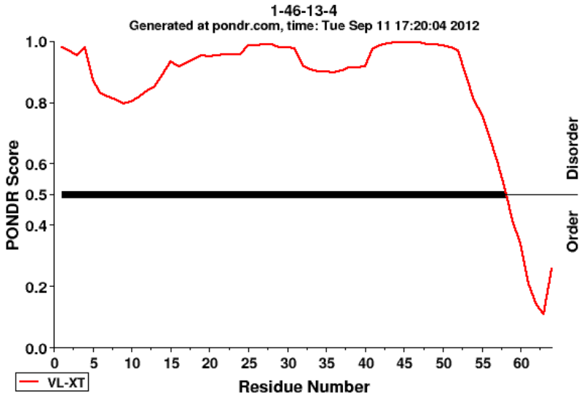

1-46-13-4
Generated at pondr.com, time: Tue Sep 11 17:20:04 2012
PONDR Score
Residue Number
Disorder
Order
VL-XT

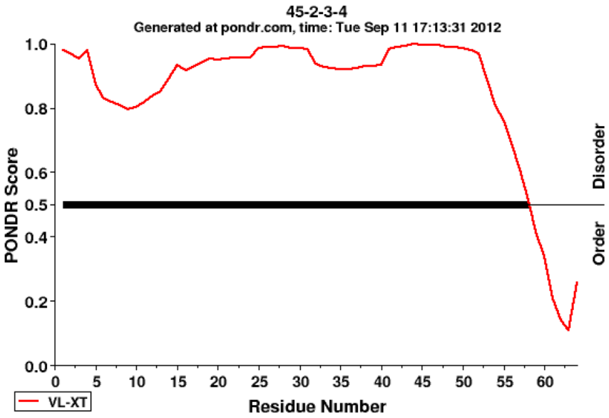

45-2-3-4
Generated at pondr.com, time: Tue Sep 11 17:13:31 2012
PONDR Score
Residue Number
Disorder
Order
VL-XT

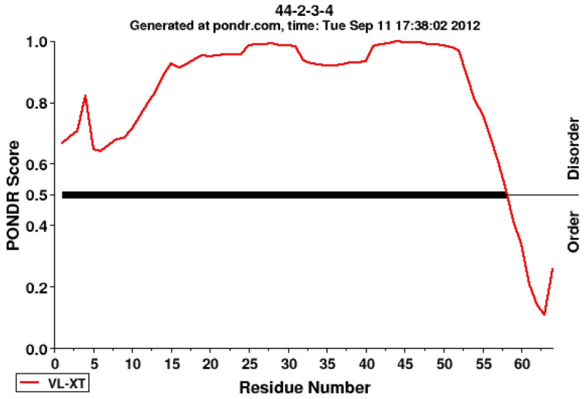

44-2-3-4
Generated at pondr.com, time: Tue Sep 11 17:38:02 2012
PONDR Score
Residue Number
Disorder
Order
VL-XT

Intrinsic protein disorder prediction for 7R haplotypes

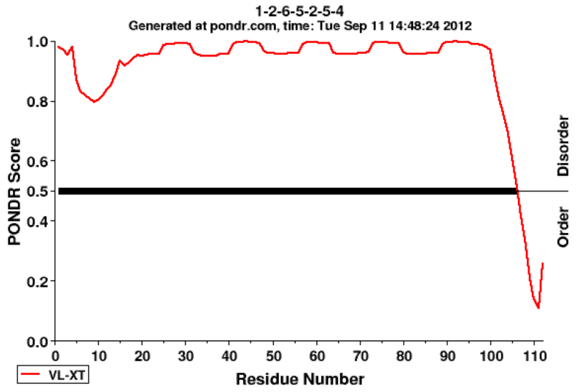

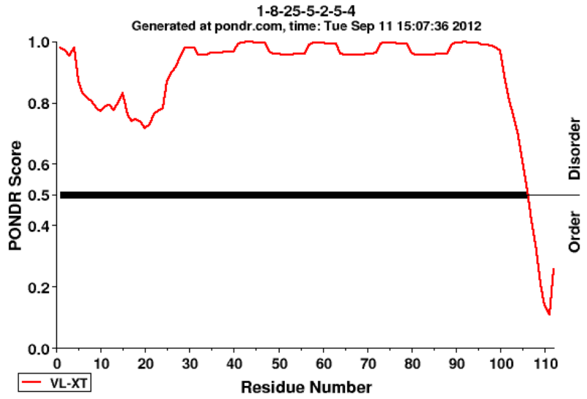

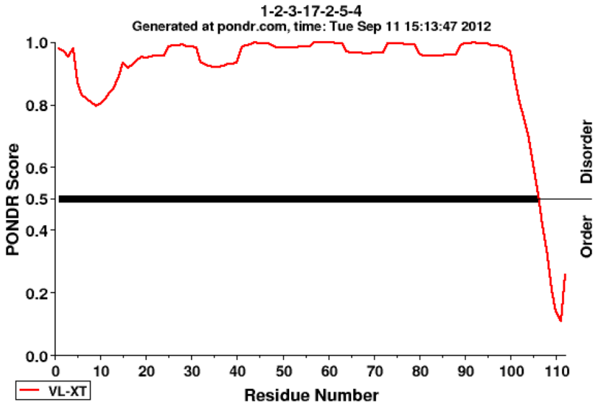

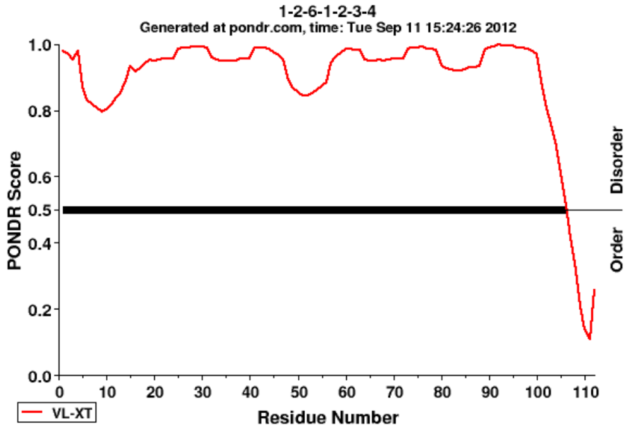

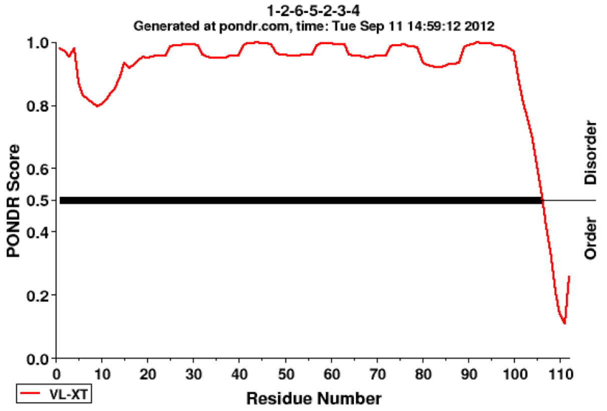

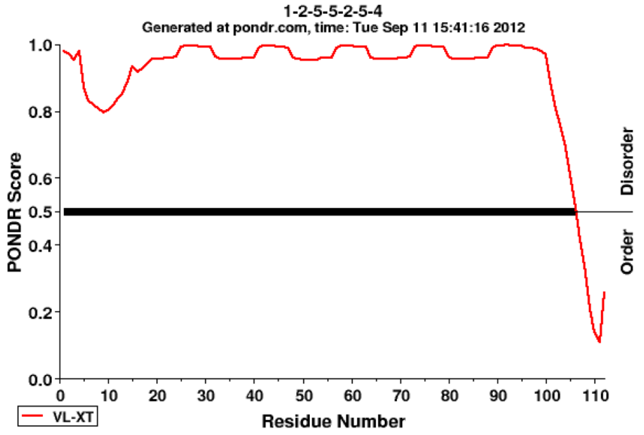

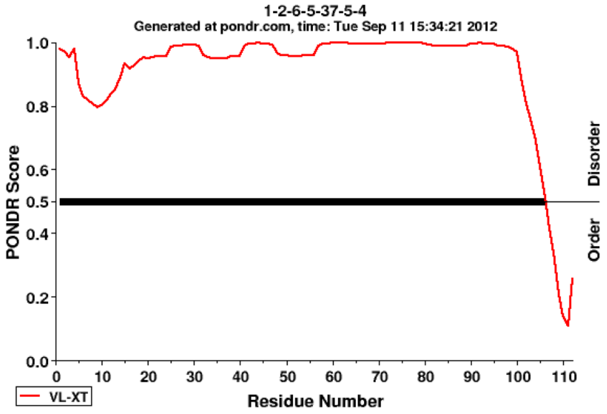

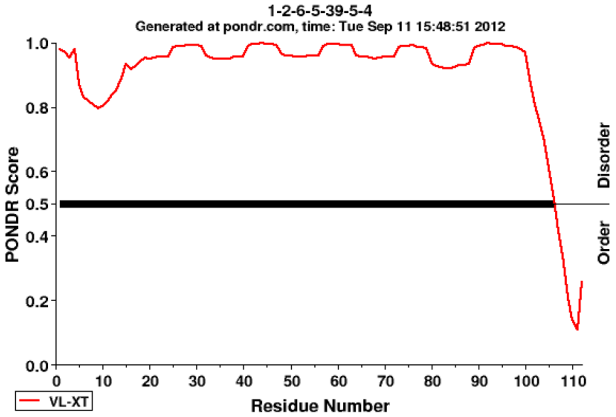

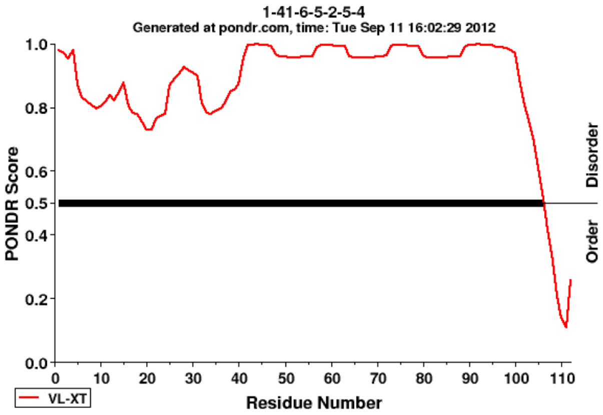
1-41-6-5-2-5-4
Generated at pondr.com, time: Tue Sep 11 16:02:29 2012
PONDR Score
Residue Number
Disorder
Order
VL-XT

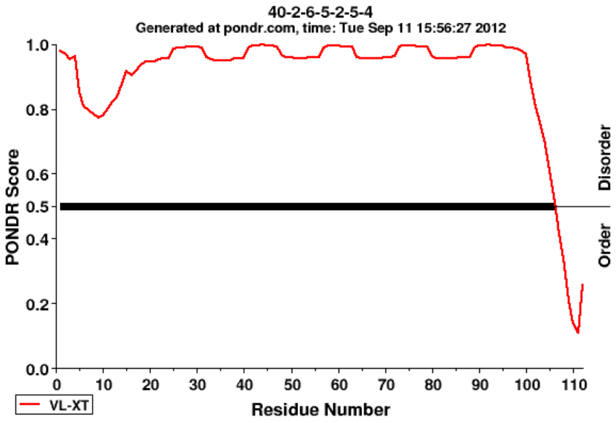
40-2-6-5-2-5-4
Generated at pondr.com, time: Tue Sep 11 15:56:27 2012
PONDR Score
Residue Number
Disorder
Order
VL-XT
