# Supplementary figures and images for: DRD4 Rare Variants in Attention-Deficit/Hyperactivity Disorder (ADHD): Further Evidence from a Birth Cohort Study
Source: PLoS One. 2013 Dec 31;8(12):e85164. doi: 10.1371/journal.pone.0085164 (PMC3877354; doi:10.1371/journal.pone.0085164)

## Study Flow Chart

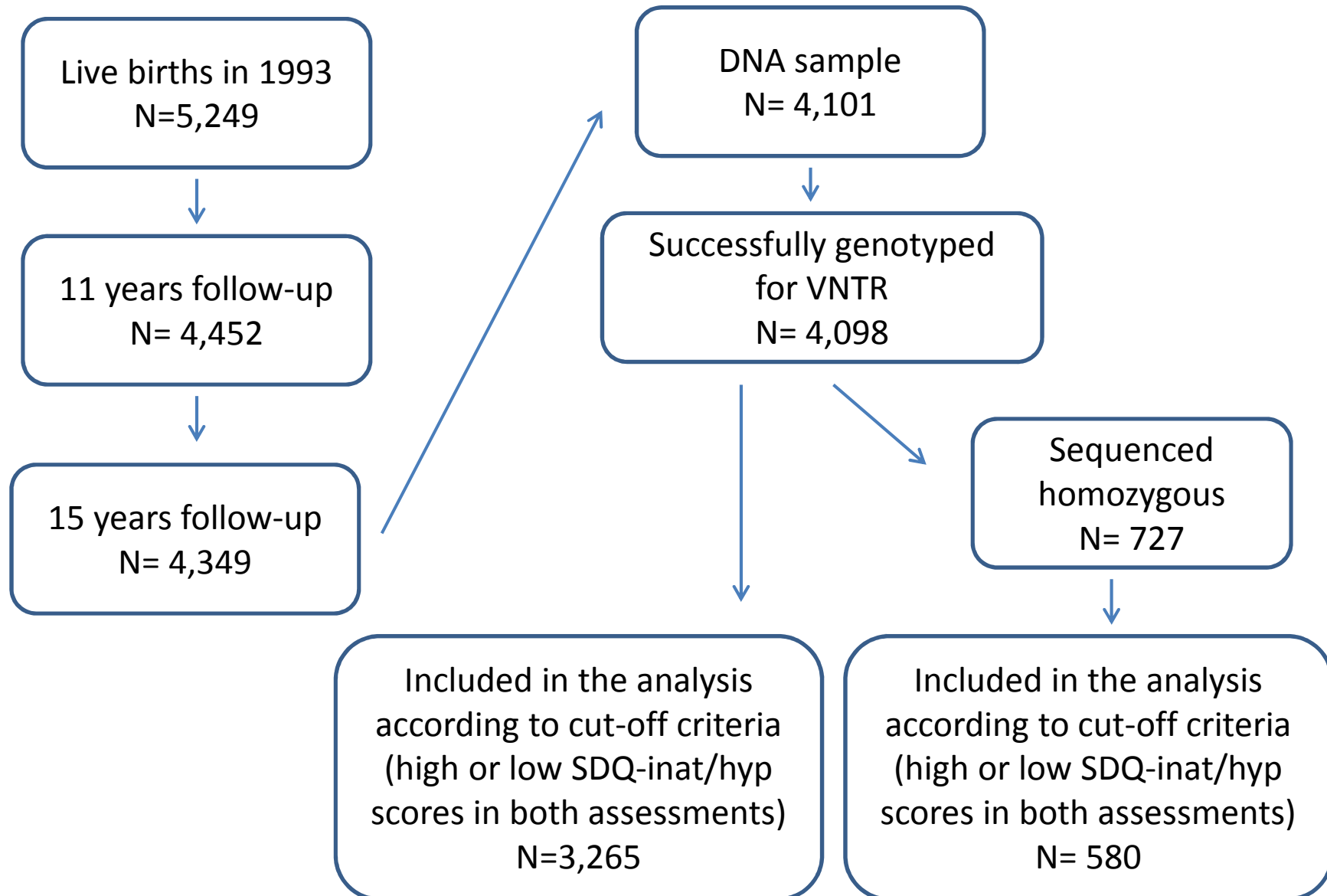

Supplement: Figure S3 — Study flow chart specifying the sample size for each analysis. (PDF) [file pone.0085164.s003.pdf]
