# Supplementary material for: DRD4 Rare Variants in Attention-Deficit/Hyperactivity Disorder (ADHD): Further Evidence from a Birth Cohort Study
Source: PLoS One. 2013 Dec 31;8(12):e85164. doi: 10.1371/journal.pone.0085164 (PMC3877354; doi:10.1371/journal.pone.0085164)
Supplement: Table S1 — Frequencies of genotypes considering allele length observed in total sample, in low-score and high-score groups. (DOCX) [file pone.0085164.s004.docx]

Table S1: Frequencies of genotypes considering allele length observed in total sample, in low-score and high-score groups.

| **Genotype** | **Population sample** | **Low-score group** | **High-score group** |
| --- | --- | --- | --- |
| 2.2R | 32 (0.8%) | 26 (0.9%) | 0 (0.0%) |
| 2.4R | 484 (11.8%) | 338 (11.6%) | 43 (12.7%) |
| 2.7R | 126 (3.1%) | 85 (2.9%) | 9 (2.7%) |
| 4.4R | 1,745 (42.6%) | 1,256 (42.9%) | 142 (41.9%) |
| 4.7R | 1,068 (26.1%) | 779 (26.6%) | 84 (24.8%) |
| 7.7R | 182 (4.4%) | 125 (4.3%) | 19 (5.6%) |
| Other | 461 (11.2%) | 317 (10.8%) | 42 (12.3%) |
| **Total** | **4,098 (100.0%)** | **2,926 (100.0%)** | **339 (100.0%)** |

HWE test: P > 0.05 for the population sample, low and high score groups
