# Supplementary material for: DRD4 Rare Variants in Attention-Deficit/Hyperactivity Disorder (ADHD): Further Evidence from a Birth Cohort Study
Source: PLoS One. 2013 Dec 31;8(12):e85164. doi: 10.1371/journal.pone.0085164 (PMC3877354; doi:10.1371/journal.pone.0085164)
Supplement: Table S2 — Allele frequency model for 4R and 7R. (DOCX) [file pone.0085164.s005.docx]

Table S2: Allele frequency model for 4R and 7R

| Allele | **Low-score group** | **High-score group** | **Adjusted Residual P-value** |
| --- | --- | --- | --- |
| 4R | 3850 (65.79%) | 441 (65.04%) | 0.699 |
| 7R | 1172 (20.03%) | 141 (20.80%) | 0.636 |
| Other | 830 (14.18) | 96 (14.15%) | 0.987 |
| Total (2N) | **5,852 (100.0%)** | **678 (100.0%)** |  |

Fisher Exact Test P-value: 0.884
