# Supplementary material for: DRD4 Rare Variants in Attention-Deficit/Hyperactivity Disorder (ADHD): Further Evidence from a Birth Cohort Study
Source: PLoS One. 2013 Dec 31;8(12):e85164. doi: 10.1371/journal.pone.0085164 (PMC3877354; doi:10.1371/journal.pone.0085164)
Supplement: Table S3 — Frequency of DRD4-VNTR genotypes regarding presence or absence of 7R allele length and respective estimated odds ratios (OR) for high hyperactivity and inattention scores in the sequenced subsample. (DOC) [file pone.0085164.s006.doc]

Table S3: Frequency of DRD4-VNTR genotypes regarding presence or absence of 7R allele length and respective estimated odds ratios (OR) for high hyperactivity and inattention scores in the sequenced subsample.

| Genotype | Low-score group N (%) | High-score group  N (%) | OR (95% CI) | P-value |
| --- | --- | --- | --- | --- |
| 7R carriers | 119 (23.20%) | 19 (28.36%) | 1.438 (0.805-2.565) | 0.218 |
| Others | 394(76.80%) | 48 (71.64%) | 1 | -- |

The OR, 95% CI and P value were calculated by multivariate logistic regression adjusted for gender and ethnicity.
